# Feline immunodeficiency virus in puma: Estimation of force of infection reveals insights into transmission

**DOI:** 10.1002/ece3.5584

**Published:** 2019-09-26

**Authors:** Jennifer J. H. Reynolds, Scott Carver, Mark W. Cunningham, Ken A. Logan, Winston Vickers, Kevin R. Crooks, Sue VandeWoude, Meggan E. Craft

**Affiliations:** ^1^ Department of Veterinary Population Medicine University of Minnesota St Paul MN USA; ^2^ School of Biological Sciences University of Tasmania Hobart Tas. Australia; ^3^ Florida Fish and Wildlife Conservation Commission Gainesville FL USA; ^4^ Colorado Parks and Wildlife Montrose CO USA; ^5^ Wildlife Health Center University of California Davis Davis CA USA; ^6^ Department of Fish, Wildlife, and Conservation Biology Colorado State University Fort Collins CO USA; ^7^ Department of Microbiology, Immunology, and Pathology Colorado State University Fort Collins CO USA

**Keywords:** age‐prevalence data, cougar, disease‐induced mortality, feline immunodeficiency virus, force of infection modeling, pathogen transmission, puma

## Abstract

1. Determining parameters that govern pathogen transmission (such as the force of infection, FOI), and pathogen impacts on morbidity and mortality, is exceptionally challenging for wildlife. Vital parameters can vary, for example across host populations, between sexes and within an individual's lifetime.
2. Feline immunodeficiency virus (FIV) is a lentivirus affecting domestic and wild cat species, forming species‐specific viral–host associations. FIV infection is common in populations of puma (*Puma concolor*), yet uncertainty remains over transmission parameters and the significance of FIV infection for puma mortality. In this study, the age‐specific FOI of FIV in pumas was estimated from prevalence data, and the evidence for disease‐associated mortality was assessed.
3. We fitted candidate models to FIV prevalence data and adopted a maximum likelihood method to estimate parameter values in each model. The models with the best fit were determined to infer the most likely FOI curves. We applied this strategy for female and male pumas from California, Colorado, and Florida.
4. When splitting the data by sex and area, our FOI modeling revealed no evidence of disease‐associated mortality in any population. Both sex and location were found to influence the FOI, which was generally higher for male pumas than for females. For female pumas at all sites, and male pumas from California and Colorado, the FOI did not vary with puma age, implying FIV transmission can happen throughout life; this result supports the idea that transmission can occur from mothers to cubs and also throughout adult life. For Florida males, the FOI was a decreasing function of puma age, indicating an increased risk of infection in the early years, and a decreased risk at older ages.
5. This research provides critical insight into pathogen transmission and impact in a secretive and solitary carnivore. Our findings shed light on the debate on whether FIV causes mortality in wild felids like puma, and our approach may be adopted for other diseases and species. The methodology we present can be used for identifying likely transmission routes of a pathogen and also estimating any disease‐associated mortality, both of which can be difficult to establish for wildlife diseases in particular.

## Graphical abstract

Age‐prevalence data are used to estimate the force of infection (FOI) of feline immunodeficiency virus (FIV) in puma. FIV is widespread in puma populations, yet its impact on puma mortality and its transmission routes are unclear. Our findings yield insights into the transmission of FIV and show no evidence of disease‐associated mortality. The FOI is shown to vary with puma sex and geographic location.

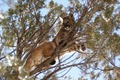

## 1 INTRODUCTION

Fundamental to understanding dynamics of infectious disease in populations is an appreciation of the force of infection (FOI), defined as the per capita rate at which susceptible individuals acquire infection (Anderson & May, 1991; Muench, 1959). This is driven by two critical components: the rate of contact and probability of transmission given a contact. It also depends on the number of infectious individuals in the population. In general, precise measurements of pathogen transmission are challenging to establish because of the considerable difficulties associated with identifying the nature of a potential contact and the probability of infection (McCallum, Barlow, & Hone, 2001). Additionally, heterogeneities in transmission can arise, for example due to age‐ or sex‐specific differences among individuals, or in different geographic locations. Estimating the FOI can elucidate the transmission process and offer insight into how disease is spread; this can help us better manage disease by understanding the subpopulations most at risk of infection.

One way to estimate the FOI is to use observed age‐prevalence data or the proportion of individuals of each age that test positive for a specific pathogen (Hens et al., 2010). Age‐specific FOI estimates show the risk of infection with respect to age: the risk of infection could be constant across a lifetime, could increase or decrease with age, or could peak or dip at a certain age. Age‐specific FOI estimates can therefore help identify age classes most at risk of becoming infected and may highlight likely transmission routes. Using age‐prevalence data to estimate the FOI has the additional advantage of enabling the disease‐associated mortality rate to be estimated, hence establishing a more complete picture of the foothold of a disease on a population. Disease‐associated mortality is the increase in mortality hazard resulting from becoming infected, relative to an animal that remains susceptible.

Lentiviruses, a genus of the Retroviridae, cause persistent infection that results in chronic and sometimes deadly disease. These viruses are widespread, forming species‐specific infections in primates, felids, ungulates, and humans (e.g., human immunodeficiency virus or HIV). Lentiviral transmission is dominated by sexual contacts and blood transfusions for humans, and through aggressive encounters for wildlife. HIV infection results in a gradual depletion of CD4 T‐lymphocytes, eventually compromising the host immune system and ultimately resulting in acquired immunodeficiency syndrome (AIDS). Feline immunodeficiency virus (FIV) is a feline analogue of HIV, and both domestic and wild cats can become infected with species‐specific forms (Troyer et al., 2005). Infection by FIV in felids seems to result in a continuum of disease effects. Domestic cats are affected by FIV*_Fca_*, which in some cases can result in disease symptoms including immunodepression, similar to HIV infection in humans (Bȩczkowski et al., 2015; Diehl, Mathiason‐Dubard, O'Neil, Obert, & Hoover, 1995; Pedersen, Ho, Brown, & Yamamoto, 1987). In some instances, FIV*_Fca_* infection in domestic cats has been reported to lead to mortality (Diehl et al., 1995; Pedersen et al., 1987), although not in other studies (Addie et al., 2000; Hofmann‐Lehmann, Holznagel, Ossent, & Lutz, 1997; Liem, Dhand, Pepper, Barrs, & Beatty, 2013). African lions infected with their species‐specific form (FIV*_Ple_*) have been shown to display significant CD4 depletion (Bull et al., 2003; Roelke et al., 2006) and other adverse clinical and immunological outcomes that parallel those caused by lentivirus infection in humans and domestic cats (Roelke et al., 2009). However, there is evidence to suggest that there are also infected lions without overt disease that can have normal life spans and reproduce successfully (Carpenter & O'Brien, 1995; Hofmann‐Lehmann et al., 1996; Packer et al., 1999).

The puma (*Puma concolor*; also called cougar, mountain lion or panther) is a large felid in the Americas that harbors a distinct FIV strain adapted to puma (FIV*_Pco_*; Lee et al., 2017), with unknown FOI and largely unknown pathology to the host. Pumas are currently found throughout most of South and Central America and in the western part of North America, as well as in a relictual population in southern Florida (Hansen, 1992). FIV*_Pco_*, hereafter called FIV, is common in pumas across their geographic range (Bevins et al., 2012; Carpenter et al., 1996; Evermann, Foreyt, Hall, & McKeirnan, 1997), with up to 60% of individuals in a population infected (Biek et al., 2003; Biek, Ruth, Murphy, Anderson, & Poss, 2006; Carpenter et al., 1996; Carver et al., 2016; Roelke et al., 1993). Despite its endemic status, the effects of the virus on the health of pumas remain undefined. FIV infection persists throughout the lifetime of the animal (VandeWoude & Apetrei, 2006), and infected pumas exhibit a decline in lymphocytes (Roelke et al., 2006), suggesting immune perturbations are induced during infection similar to other lentiviral infections. Published accounts indicate that FIV‐infected puma do not have increased co‐exposure to other pathogens (Biek et al., 2006; Gilbertson et al., 2016). A study of pumas from Montana and Wyoming found no evidence for an overall reduction in survival due to infection when accounting for other sources of demographic variation (age, sex and population); however, results from stochastic simulations in this study (Biek et al., 2006) indicated that only larger reductions in annual survival (>20%) could be excluded with confidence. Therefore, the impact of FIV infection on puma mortality is still uncertain.

This study aims to address the uncertainty over whether FIV affects puma mortality, and to identify the factors that influence the FOI in order to establish which subpopulations are most at risk of infection. This may also increase the understanding of the disease in its other hosts. We use age‐prevalence data (based on serology, a marker of infection owing to FIV being chronic and lifelong) to estimate the age‐specific FOI of puma FIV. Our specific goals are to determine (a) if and how the FOI depends on age; (b) if the FOI is sex‐specific and if it depends on location; and (c) if there is evidence of disease‐associated mortality. Because there is a strong positive relationship between puma age and FIV prevalence (Bevins et al., 2012; Biek et al., 2003; Carver et al., 2016; Lewis et al., 2017; Roelke et al., 2006), it can be difficult to determine the effects of FIV on puma; factors that may seem to be increasing with FIV infection may instead be increasing as a result of the pumas aging. Age‐prevalence data alone do not accurately measure a disease's impact on a population, as it is subject to the biasing effects of disease‐associated mortality. The FOI is a preferred metric to evaluate a disease's foothold (Heisey, Joly, & Messier, 2006), as it adjusts for disease‐associated mortality and can show directly how the risk of infection varies with age.

To better understand the risk of FIV infection for puma, it is important to consider the natural history of the species. Pumas are apex predators that exist at low population densities and are considered solitary, except for mothers and cubs in juvenile dependency (Logan & Sweanor, 2001). Pumas can live for up to 15 years and sometimes even 20 years. Contact between adults occurs mainly through mating or during territorial fights among males. In addition, a recent study shows that there is more contact among adult pumas at food resources than previously thought and demonstrates the existence of sophisticated social behaviors such as reciprocity (Elbroch, Levy, Lubell, Quigley, & Caragiulo, 2017). Sexual maturity is reached after 2 years or sometimes earlier for female pumas. Females give birth typically at around 27–29 months old (Ashman, Christensen, Hess, Tsukamoto, & Wickersham, 1983; Logan & Sweanor, 2001), with an average litter size of 2 or 3 (Beier, Riley, & Sauvajot, 2010). Kittens are weaned from around two months old. They remain with their mother until becoming independent after 6 months (but usually between 10 and 20 months of age); they disperse out of the mother's home range shortly thereafter (Maehr, Land, Shindle, Bass, & Hoctor, 2002; Sweanor, Logan, & Hornocker, 2000). Almost all male offspring disperse out of the natal population, whereas 50%–80% of female offspring remain (Sweanor et al., 2000). Home ranges of adult male pumas are larger, with less overlap among them, compared to those of females (Beier et al., 2010). Fights between adult males and younger male or female pumas are common (Beier et al., 2010) with male pumas having more aggressive interactions than females.

Research shows region‐specific variation in FIV‐environmental associations for puma, suggesting the FOI may differ among geographic locations and environments (Carver et al., 2016). To determine if the FOI displays such variation, we compare puma in three different locations with contrasting landscape fragmentation and management (southern California, Colorado, and Florida). Southern California puma persist in highly fragmented urban populations; Colorado puma exist in more intact habitat and in some areas are recreationally hunted; while Florida panthers are endangered and have had their population genetically supplemented from Texas puma. Sex is another factor that has been reported to influence FIV prevalence, with male pumas generally more likely to be infected with FIV than females (Bevins et al., 2012). Due to this, we compare the FOI of females and males to determine if this difference in FIV prevalence between the sexes is reflected in the FOI.

In order to estimate the FOI, we test a range of different candidate FOI models. Transmission of FIV occurs via direct contact between individual pumas, although the exact route(s) of transmission are not clear. Our candidate models are flexible and represent a variety of combinations of possible direct transmission modes. If the main transmission routes are aggressive interactions involving biting/fighting (Pontier, Fromont, Courchamp, Artois, & Yoccoz, 1998) and mating (Biek et al., 2003; VandeWoude & Apetrei, 2006), we might expect adults to have a higher FOI than juveniles. This hypothesis is supported by research suggesting that transmission is more common after sexual maturity (as reviewed in VandeWoude & Apetrei, 2006). We might also expect to see a higher FOI for males than females, possibly as a result of males having a higher frequency of aggressive interactions (Beier et al., 2010). Alternatively, if mother to kitten transmission is the main route (perhaps via saliva during grooming or feeding together) we might expect to see a higher FOI in younger animals, prior to independence of offspring. Closely related virus sequences have been isolated from mother–cub puma pairs, suggesting that transmission can occur via contact between mother and kitten (Biek et al., 2003; Carpenter et al., 1996), although this has been considered to occur only rarely (VandeWoude & Apetrei, 2006). Our estimation of the FOI can thus potentially yield important insights into the likelihood of different transmission routes of FIV between pumas.

## 2 METHODS

### 2.1 Data collection

Pumas from three study sites spanning coastal southern California, Colorado, and Florida were sampled and their FIV*_Pco_* status assessed. Specifically, the three study sites were Orange County in California, Western Slope in Colorado, and southern Florida; descriptions and details of these sites are given in Carver et al. (2016). Pumas were captured and biological samples collected from 1999 to 2013 as part of ongoing research (Bevins et al., 2012; Carver et al., 2016; Troyer et al., 2014). Exposure to FIV was estimated by measuring serum antibodies according to protocols previously described (Bevins et al., 2012; Carver et al., 2016). Puma sex and age were also recorded at capture. Some of the younger pumas in the study were of known age, that is, the approximate birth date was known. For all other animals, age was estimated using a variety of different methods (including those described in Ashman et al., 1983; Laundré, Hernndez, Streubel, Altendorf, & Lpez Gonzlez, 2000). This involved assessment of size, weight, dental characteristics, and morphological features (e.g., tail length). Comparing animals to those of known age was useful in deriving age estimates. If the same animal was captured on more than one occasion, we used the result from the most recent capture in our analysis. There were less results for older pumas, so we opted to include the most recent capture in order to maximize the data for older pumas. Kittens were excluded from the study if they tested positive but were younger than 6 months of age (due to the possibility of maternal antibodies being present). There were a total of 209 pumas in the data set (109 females and 100 males).

Data were grouped into bins according to puma age. The age bins applied were 0.5, 1, 2, 3–4, 5–6, 7–8, 9+ years, or more formally [0, 0.75), [0.75, 1.5), [1.5, 2.5), [2.5, 4.5), [4.5, 6.5), [6.5, 8.5), [8.5, 13.5] years. Age bins were used rather than separating the data into single year groups, due to the age estimation process not being completely accurate, and to increase the number of animals in each data group. We used broader bins as puma age increased, both because age accuracy tends to be more uncertain as pumas get older, and because there were fewer older pumas in the data set. The age bins were also selected with consideration to the life history of the puma. In addition to analyzing all data together, we separated the data according to sex and then area and sex.

We calculated the prevalence of FIV (number of infected animals/total number of animals) in the total population and in all subpopulations. We also derived 95% confidence intervals of the prevalence values, using the *binom.test* function in R (R Core Team, 2013; http://www.R-project.org/). This function employs the Clopper–Pearson method for confidence intervals for a binomial proportion.

### 2.2 Fitting candidate models to data

We estimate the FOI by testing and comparing a variety of different candidate models, shown in Table 1. We denote the disease‐associated mortality by the parameter *μ* and the FOI by *λ*. In addition to a constant FOI, we consider a variety of age‐varying models: a Weibull model, a log‐logistic model, and the polynomial FOI of Grenfell and Anderson (1985), which has the flexibility to fit many shaped curves. Considering this range of candidate models ensures that we test a full range of possible shapes for the FOI (including constant, decreasing, increasing curves, and curves with peaks) and hence consider a wide variety of possible different means of FIV transmission. From each candidate FOI, one can derive the predicted age‐specific disease prevalence, for two different cases: with and without disease‐associated mortality. As an example, we present the derivation of one of the age‐specific disease prevalence functions (for constant *λ* with *μ* ≠ 0) in Appendix A. We denote puma age by *a*, so when the FOI depends on age it is written *λ*(*a*). We assume that when *a* = 0, the FIV prevalence is also 0. The units of age *a* are years, and the units of rate *μ* are per year.

**Table 1:** Force of infection models and their corresponding age‐specific disease prevalence functions

Force of infection *λ*(*a*)	Age‐specific disease prevalence	
	*μ* = 0	*μ* ≠ 0
Constant		
*λ*(*a*) = *λ*	1 − *e* ^−^ *^λa^*	$\frac{\lambda [1-e^{(\mu -\lambda )a}]}{\lambda -\mu e^{(\mu -\lambda )a}}$
Weibull		
$\lambda \left(a\right)=\beta \gamma a^{\gamma -1}$	$1-e^{-\beta a^{\gamma }}$	No explicit solution
Log‐logistic		
$\lambda \left(a\right)=\frac{\beta \gamma {\left(\beta a\right)}^{\gamma -1}}{1+{\left(\beta a\right)}^{\gamma }}$	$1-\frac{1}{1+{\left(\beta a\right)}^{\gamma }}$	No explicit solution
Polynomial		
$\lambda \left(a\right)=\sum_{i=0}^{k}b_{i}a^{i}$	$1-e^{-\sum_{i=0}^{k}\frac{b_{i}a^{i+1}}{i+1}}$	No explicit solution

Note

The disease‐associated mortality rate is *μ*, and puma age is *a*. Parameters *β*, *γ*, *b*, and *k* describe the shape of the different models and are to be estimated. Parameter *k* must take an integer value.

Once the predicted age‐specific disease prevalence functions were derived, these were compared and fitted to the FIV age‐prevalence data. We used maximum likelihood to fit to the data, by minimizing the negative log‐likelihood. This optimization was performed using a sequential quadratic programming method in GNU Octave (version 4.0.0; http://www.gnu.org/software/octave; Eaton, Bateman, Hauberg, & Wehbring, 2015). We used the solver *sqp*, which performs general nonlinear minimization. For each age bin, we calculated the negative log‐likelihood of the model prediction of disease prevalence. This factored in the sample size of each age bin. The sum of these individual negative log‐likelihoods is the metric that was minimized. (Note that minimization of negative log‐likelihood is equivalent to the maximization of the log‐likelihood.) The GNU Octave routine (see Appendix B for code) requires an initial guess for the parameter values, plus lower and upper bounds, to be provided. It returns the parameter values that minimize the total negative log‐likelihood. Table 2 shows the parameters to estimate for each candidate model. All parameters were assumed non‐negative and *γ* > 1. Parameter *k* takes an integer value.

**Table 2:** Parameters to estimate for each candidate model during the process of fitting the age‐specific disease prevalence function to the FIV prevalence data

Candidate model	Parameters to estimate
Constant, *μ* = 0	*λ*
Constant, *μ* ≠ 0	*λ*, *μ*
Weibull, *μ* = 0	*β*, *γ*
Weibull, *μ* ≠ 0	*β*, *γ*, *μ*
Log‐logistic, *μ* = 0	*β*, *γ*
Log‐logistic, *μ* ≠ 0	*β*, *γ*, *μ*
Polynomial, *μ* = 0	*b*_0…_ *_k_*, *k*
Polynomial, *μ* ≠ 0	*b*_0…_ *_k_*, *k*, *μ*

For the cases without an explicit solution to fit (see Table 1), we developed a routine in GNU Octave to find the age‐specific disease prevalence using numerical methods and then fit to the data. For example, for the Weibull *μ* ≠ 0 model, the equations to be solved are:

$$\frac{dS}{da}=-\beta \gamma a^{\gamma -1}S$$

$$\frac{dI}{da}=\beta \gamma a^{\gamma -1}S-\mu I$$

with *S* the proportion of susceptible pumas, *I* the proportion of infected pumas, and parameters *β* and *γ* describing the shape of the Weibull model. We used an ordinary differential equation solver (*lsode*) and then taking

$$\frac{I}{I+S}$$

yielded the disease prevalence to fit to the data. Solving the equations was carried out within the optimization procedure.

### 2.3 Comparing the candidate models

We used second order Akaike information criterion or AICc (Burnham & Anderson, 2002) to compare the fit of each of the candidate models. This takes into account the sample size and the number of parameters involved in each model and penalizes the fit accordingly. For the best models (i.e., with the lowest AICc values), we calculated the *χ* ^2^ statistic and corresponding P (probability) value to assess model fit. The smaller the P value, the stronger the evidence against the hypothesis that the data fit the model. In addition, we found 95% confidence intervals using the method of profile likelihood (Caley & Hone, 2002; McCallum, 2000), a method for determining confidence intervals for maximum likelihood estimates, for the parameters of the best models. Pearson residuals were calculated and plotted in GNU Octave to further examine the fit of the best models.

## 3 RESULTS

### 3.1 FIV prevalence

**Figure 1:**
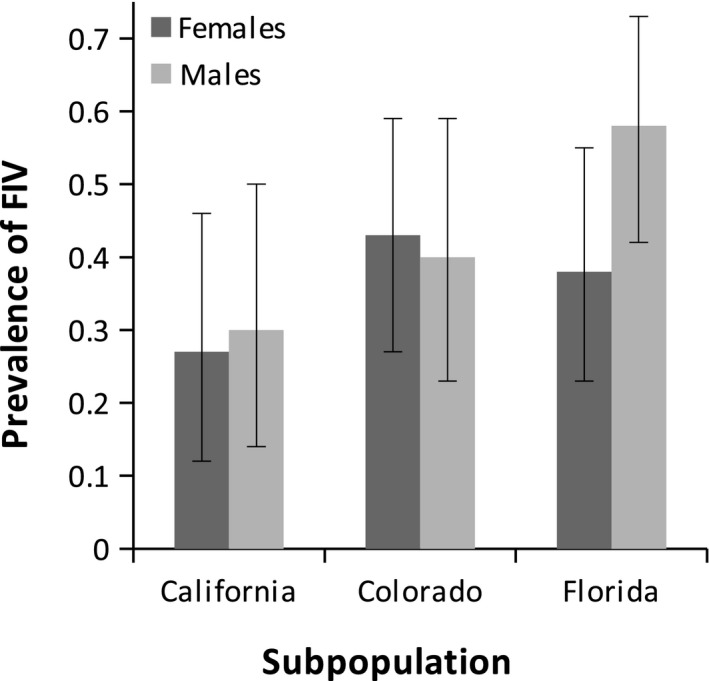
The prevalence of FIV for each site and sex. 95% confidence intervals are indicated by the black lines on each bar

**Figure 2:**
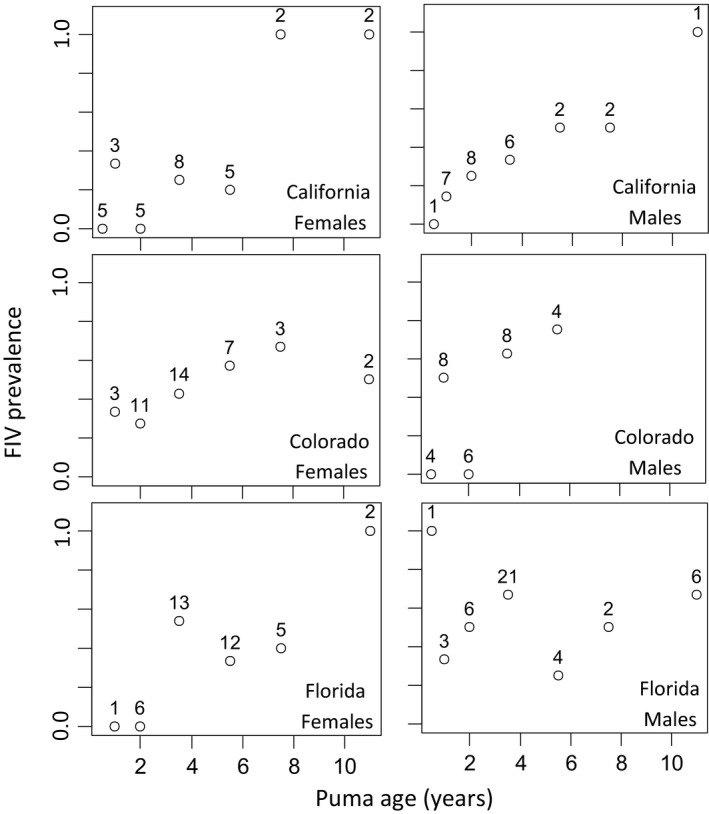
FIV age‐prevalence data for each sex and area. Ages are binned as described in Section 2, and data points are plotted at the midpoint of each age bin. The numbers above each data point indicate the number of pumas in that age bin

**Figure A1:**
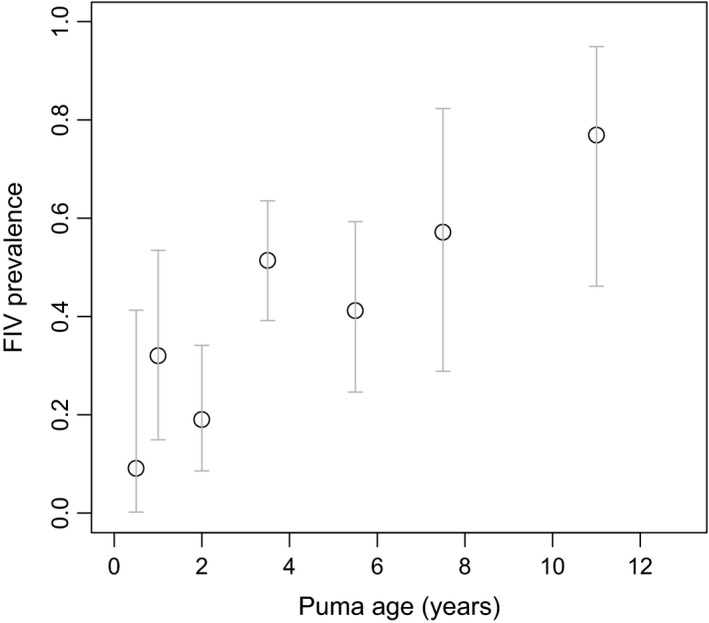
Age‐prevalence data for puma FIV, from all three areas and for both sexes. Ages are binned as described in Section 2, and data points are plotted at the midpoint of each age bin. 95% confidence intervals are indicated by the gray lines

**Figure A2:**
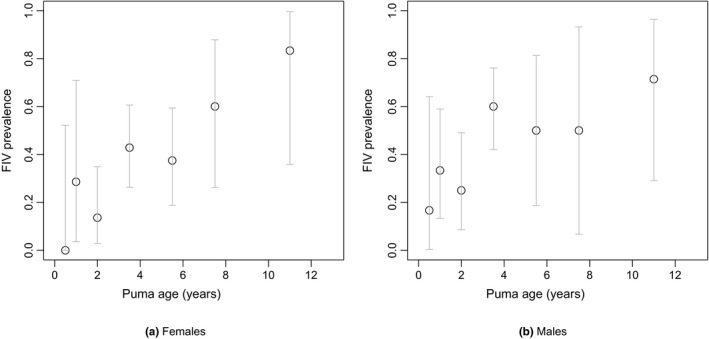
FIV age‐prevalence data separated according to puma sex. Ages are binned as described in Section 2, and data points are plotted at the midpoint of each age bin. 95% confidence intervals are indicated by the gray lines

The overall prevalence of FIV infection in our study was 0.41, with a 95% confidence interval (CI) of 0.34–0.48. Male prevalence (0.45, CI: 0.35–0.55) was higher than female prevalence (0.37, CI: 0.28–0.46). The prevalence for Florida males was the highest of all subpopulations (0.58, CI: 0.42–0.73), whereas the prevalence for California females was the lowest (0.27, CI: 0.12–0.46; Figure 1). FIV prevalence generally increased with age (see Figures A1 and A2). The age‐prevalence data separated by both sex and area are shown in Figure 2, and the numbers of puma in each age bin are given.

### 3.2 Candidate FOI models

The AICc values for the FOI models are shown for the data separated by sex and area in Table 3 (females) and Table 4 (males). AICc values for data from all sites and both sexes together, and also for data separated by sex, are given in Tables A1 and A2, respectively.

**Table 3:** AICc values for each model for data for female pumas separated according to area

Force of infection *λ*(*a*)	California		Colorado		Florida	
	AICc		AICc		AICc	
	*μ* = 0	*μ* ≠ 0	*μ* = 0	*μ* ≠ 0	*μ* = 0	*μ* ≠ 0
Females						
Constant	**29.12**	31.42	**55.00**	57.04	**50.23**	52.46
Weibull	*30.37*	32.66	58.02	59.38	52.42	54.58
Log‐logistic	31.26	33.74	57.19	59.37	52.39	54.74
Polynomial, *k* = 1	*29.93*	32.41	58.02	59.38	52.43	54.72
Polynomial, *k* = 2	31.52	33.67	60.37	61.85	54.71	57.05

Note

The lowest AICc value for each area is shown in bold, and any others that are <2 from the lowest are italicized.

**Table 4:** AICc values for each model for data for male pumas separated according to area

Force of infection *λ*(*a*)	California		Colorado		Florida	
	AICc		AICc		AICc	
	*μ* = 0	*μ* ≠ 0	*μ* = 0	*μ* ≠ 0	*μ* = 0	*μ* ≠ 0
Males						
Constant	**30.96**	33.30	**38.34**	40.61	69.74	65.97
Weibull	33.30	35.88	40.64	43.09	71.94	68.29
Log‐logistic	33.42	35.96	40.71	43.19	**63.94**	66.22
Polynomial, *k* = 1	33.28	35.67	40.64	42.86	71.94	68.29
Polynomial, *k* = 2	35.76	38.30	43.11	45.29	74.26	70.73

Note

The lowest AICc value for each area is shown in bold, and any others that are <2 from the lowest are italicized (there are no such values in this table).

**Figure 3:**
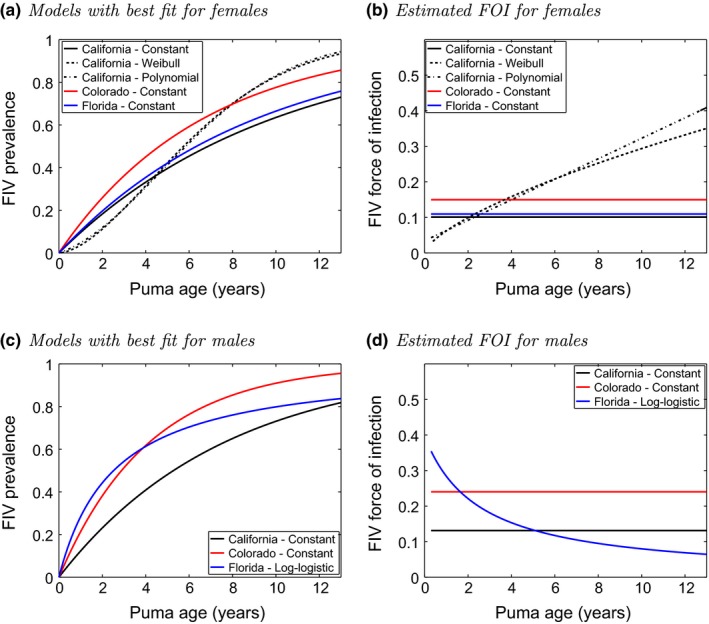
Models with best fit to the FIV prevalence data for (a) females pumas and (c) male pumas, and the corresponding estimated force of infection for (b) females and (d) males, in each of the three areas. For the female plots, the solid lines are the models with the best fit, and others are models with AICc < 2 from that of the best

For female pumas, the best fit to the data was a model with constant FOI and no disease‐associated mortality (*μ* = 0; Table A2). When we split the data by sex and area, for females in all three areas, the best models were constant FOI with no disease‐associated mortality (Table 3; Figure 3a,b). However, there were two other competitive models for the California data (Table 3; Figure 3a). These had no disease‐associated mortality and corresponded to FOI curves that increase with age (Figure 3b). For the male pumas, for California and Colorado, the best FOI models were constant with no disease‐associated mortality (Table 4; Figure 3c,d). For Florida males, the best model gave a FIV prevalence curve with a relatively sharp initial increase (Figure 3c), which corresponded to a FOI curve starting at a high value then decreasing as puma age increased (Figure 3d), and again with no disease‐associated mortality. Our findings therefore suggest that FIV in pumas has a constant FOI (i.e., the FOI does not depend on puma age) with the exception of Florida male pumas and possibly California female pumas.

**Figure A3:**
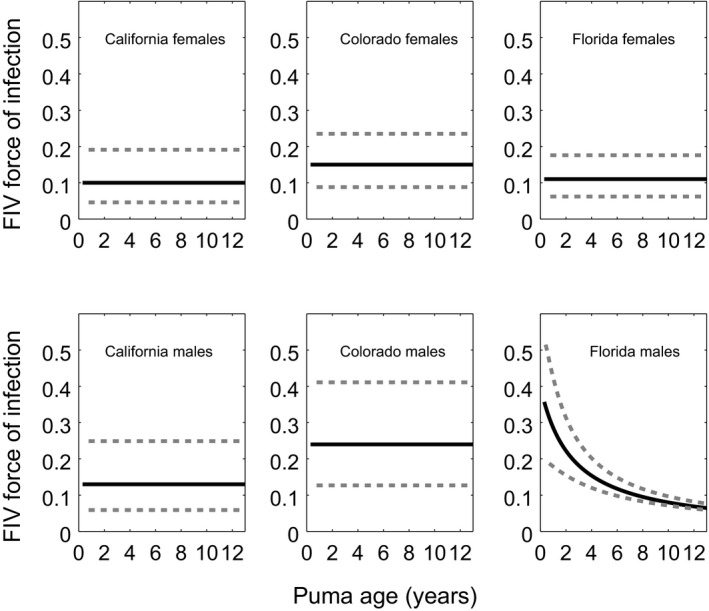
The best fit force of infection curves (solid lines) for each site and sex, with 95% confidence intervals (dashed lines)

**Figure A4:**
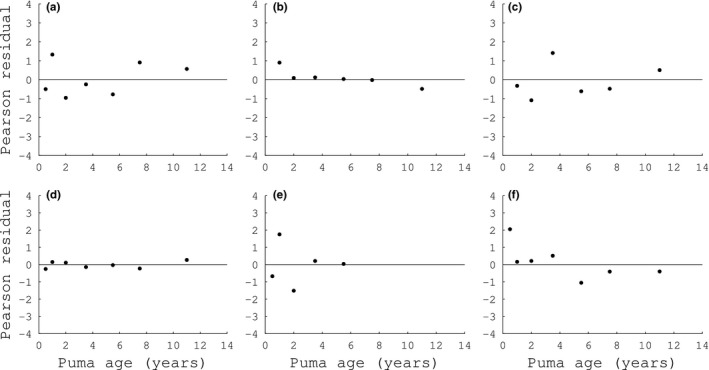
Pearson residual plots for the best models for females and males in the three different areas. (a) California females; (b) Colorado females; (c) Florida females; (d) California males; (e) Colorado males; (f) Florida males

The fit of the best models for females and males separate was better than for the data all together; the best models for females and males had P values of .66 and .85, respectively (6 degrees of freedom), compared with a P value of .33 (6 degrees of freedom) for the data all together (Table 5). (Recall that the smaller the P value, the stronger the evidence against the hypothesis that the data fits the model.) Some model fits were very good (e.g., California males; P value .9997, 6 degrees of freedom), but others were less good (e.g., Colorado males; P value .21, 4 degrees of freedom) (Table 5). Confidence intervals for the parameters of the best models and Pearson residual plots (see Figures A3 and A4) also demonstrated this. For Colorado males, there were only data for 5 age bins (out of the 7 total bins), as there were no pumas of age over 6 years (see Figure 2). This was probably due to the Colorado puma population being recreationally hunted; hunting of California and Florida populations does not occur.

**Table 5:** Estimated parameter values and statistics describing the model fit to data for the models with the best fit

Model	Estimated parameter values	*χ* ^2^ statistic	P Value	Sample size
Data all together				
**Log-logistic,** μ=0	β=0.22,γ=1.00	6.87	0.33 (6 *df*)	209
*Constant*, *μ* ≠ 0	λ=0.24,μ=0.30			
*Constant*, *μ* = 0	*λ* = 0.15			
*Polynomial*, *μ* ≠ 0	$b_{0}=0.27,\;b_{1}=0.02,\;\mu =0.50\;\left(k=1\right)$			
Females				
Constant,μ=0	*λ* = 0.12	4.12	0.66 (6 *df*)	109
Males				
**Log-logistic,** μ=0	β=0.30,γ=1.00	2.69	0.85 (6 *df*)	100
*Constant*, *μ* ≠ 0	λ=0.37,μ=0.50			
California females				
Constant,μ=0	*λ* = 0.10 (CI: 0.046–0.191)	4.74	0.58 (6 *df*)	30
*Polynomial*, *μ* = 0	$b_{0}=0.03,\;b_{1}=0.03\;\left(k=1\right)$			
*Weibull*, *μ* = 0	β=0.04,γ=1.67			
Colorado females				
Constant,μ=0	*λ* = 0.15 (CI: 0.088–0.235)	1.07	0.96 (5 *df*)	40
Florida females				
Constant,μ=0	*λ* = 0.11 (CI: 0.062–0.176)	4.13	0.53 (5 *df*)	39
California males				
Constant,μ=0	*λ* = 0.13 (CI: 0.059–0.249)	0.24	0.9997 (6 *df*)	27
Colorado males				
Constant,μ=0	*λ* = 0.24 (CI: 0.127–0.411)	5.86	0.21 (4 *df*)	30
Florida males				
**Log-logistic,** μ=0	β=0.40,γ=1.00 (CI: 0.22, 1.03 – 0.66, 1.09)	5.97	0.43 (6 *df*)	43

Abbreviations: CI, confidence interval; *df*, degrees of freedom.

For our analysis, when the same puma had been captured multiple times in the study, we only used the data from the most recent capture (as described in Section 2). When using all recapture data, the same models were found to best fit the data. Therefore, our main findings were not sensitive to our choice to include or exclude recapture data.

## 4 DISCUSSION

In this study, we estimate the force of infection (FOI) for feline immunodeficiency virus (FIV) among wild puma from three regions within the United States. We find that (a) the FOI is constant with respect to age, with the exception of Florida male pumas and possibly California female pumas; (b) the FOI is sex‐specific and is dependent upon location; and (c) there is no evidence associating FIV seropositivity with increased mortality.

Most of the best fit FOI curves are constant with respect to age, implying that transmission occurs with equal probability throughout the lifespan of the puma. This supports the idea that transmission can occur both from mothers to kittens (during postweaning period) and also throughout adult life. This is in agreement with the findings of Biek et al. (2003), but implies that transmission from mother to cub is more frequent than previous research suggests (reviewed by VandeWoude & Apetrei, 2006). Note that *in utero* transmission of FIV*_Fca_* can occur in domestic cats (O'Neil, Burkhard, Diehl, & Hoover, 1995), but we cannot draw conclusions from our study regarding the possibility of this type of transmission for puma; the youngest animal in our study was 3 months old. For Florida males, the shape of the FOI curve suggests that FIV transmission occurs at a higher rate in the first few years of life (prior to sexual maturity) than in later life, indicating that mother–cub transmission is potentially a particularly important route in this subpopulation. In addition, young males in Florida may have more aggressive encounters; in an examination of the causes of mortality in Florida puma, the majority of animals that died from intraspecific aggression were males under 3 years old (47%; Taylor, Buergelt, Roelke‐Parker, Homer, & Rotstein, 2002). It is possible that the apparently common occurrence of fighting among young Florida males may be related to a lack of unoccupied, suitable habitat for dispersing subadults (Maehr, Land, & Roelke, 1991). The risk of infection in older male pumas in Florida is relatively low.

Our results demonstrate that the FOI varies with puma sex and with geographic location. When the data are separated by sex, the fit of the best models is better than for when we used the data all together. FIV prevalence is higher for males than females in our data set overall (although not in Colorado, possibly as a result of the lack of males over 6 years old in our Colorado population), which is in agreement with other studies (Bevins et al., 2012; Biek et al., 2006) and is also consistent with previous research in domestic cats (Pontier et al., 1998). In each of our three study areas, males have a higher FOI than females, except for older Florida males. This higher risk of infection for males is probably as a result of males having more aggressive contacts (e.g., territorial fights) than females (Beier et al., 2010; Logan & Sweanor, 2001). California has the lowest FIV prevalence and the lowest FOI (except for older Florida males); this is possibly due to anthropogenic factors in this area (e.g., habitat fragmentation) influencing the frequency of contacts that may occur in less disturbed habitats (Hess, 1996). Using an agent‐based modeling approach in another wild felid (bobcat, *Lynx rufus*), Tracey, Bevins, VandeWoude, and Crooks (2014) found that generally pathogen transmission within habitat patches was lower, and between‐patch transmission was higher, in more fragmented landscapes. They also concluded that overall disease prevalence typically was lower in more fragmented landscapes, in accordance with our findings for California. It should be noted that the effects of habitat fragmentation on transmission are complicated and dependent on interactions with animal movement behavior and epidemiology (White, Forester, & Craft, 2018).

When splitting the data by sex and area, there is no evidence for disease‐associated mortality. This is supported by all the best fit models and any other competitive models. Thus, puma in our study do not appear to suffer marked mortality effects from FIV, despite the known immunosuppressive consequences of lentiviruses in humans and some cat populations. There is both theoretical and empirical evidence that argues that endemic parasites (i.e., those that are permanently maintained) can have important effects on their host (Anderson & May, 1978; Scott, 1988; Tompkins et al., 2002). For example, studies have demonstrated that parasites, including viruses, can significantly affect wildlife mortality and shape population dynamics (Cavanagh et al., 2004; Hudson, Dobson, & Newborn, 1998; Telfer et al., 2002; Tompkins & Begon, 1999). An opposing notion is that endemic parasites may have little effect on their host because parasite fitness would suffer if parasites measurably reduce host life expectancy (Lack, 1954). Our lack of evidence for FIV‐induced mortality in puma supports this and fits a theory of a long history of coevolutionary association between FIV‐like viruses and wild feline hosts (Carpenter et al., 1996; Carpenter & O'Brien, 1995), or a lack of virulence in the FIV strain adapted to puma. Time post‐infection to disease effects could also be a factor governing the impacts of FIV; perhaps the normal lifespan of the puma is relatively short compared to the time taken for the disease effects to become apparent. A possibility is that disease‐associated mortality may be dependent on time since infection, so that the disease becomes more lethal the longer an animal is infected. A potential avenue for further research would be to test a model with a disease‐associated mortality term that increases with puma age.

While we did not detect evidence of disease‐associated mortality, FIV may be having other effects on infected pumas. For example, infection may lower fecundity; Biek et al. (2006) found a consistent but nonsignificant trend toward poorer reproductive performance in FIV‐infected females. In addition, it is possible that infection with FIV may mean that animals are at a higher risk of contracting other diseases, for example feline parvovirus or feline leukemia virus (FeLV; Cunningham et al., 2008). However, data collected from 207 pumas indicated no serological evidence for a higher probability of secondary infections associated with FIV (Biek et al., 2006). Indeed, some reported co‐infection relationships may simply reflect behavior phenotypes of hosts, such that some individuals are more likely to be exposed to multiple pathogens, rather than owing to pathogen–pathogen interactions (Carver et al., 2015). An additional complication is that FIV may influence the impact of other infections rather than (or as well as) affecting exposure rates, yet the serology data is only indicative of exposure. Further examination of co‐infection patterns in puma would be a valuable direction for future research.

The FOI has been calculated for several other wildlife infections and host species. Caley and Hone (2002) estimated the FOI of *Mycobacterium bovis* in feral ferrets in New Zealand, and this enabled them to conclude that transmission likely occurs from the ingestion of infected material from the age of weaning. In addition, a strong age dependency in the FOI of *Mycobacterium bovis* in bison in Canada has been demonstrated, as well as disease‐associated mortality (Heisey et al., 2006). Like in our study, the FOI was found to be higher in males than in females for both these systems (Caley & Hone, 2002; Heisey et al., 2006). In an investigation into the FOI of *Mycoplasma agassizii* in gopher tortoises in Florida, sites with high seroprevalence were found to have substantially higher FOI than low seroprevalence sites (Ozgul, Oli, Bolker, & Perez‐Heydrich, 2009). The FOI can also be seasonal in some cases (Cattadori, Boag, Bjornstad, Cornell, & Hudson, 2005).

In North America, sympatry is frequently exhibited between puma and bobcats, and also between these two wild felids and domestic cats around the interface of natural and anthropogenic landscapes (Carver et al., 2012; Crooks, 2002; Ordeñana et al., 2010). Cross‐species transmission of the bobcat FIV subtype has occurred from bobcats to puma (Franklin et al., 2007; Lee et al., 2017), and of FeLV from domestic cats to puma (Brown et al., 2008). These transmission events may occur as a consequence of puma predation on bobcats and domestic cats, situations similar to that which fostered transmission of simian immunodeficiency virus (SIV) to humans (becoming HIV). Studying the FOI of FIV leads to an improved understanding of disease transmission in these feline species, and of lentiviral transmission in general (e.g., SIV and HIV) through potential parallels. Investigating the FOI in reservoir hosts may also help identify which age class of individuals are most likely to contribute to spillover events, and estimating the FOI in newly established host species could inform mitigation strategies, such as targeted vaccination of particular age classes.

A limitation of our method is that for many wildlife species, it is hard to accurately estimate the age of adults and it might be challenging to acquire adequate samples from different age groups, particularly from the young. In addition, splitting the data by both sex and area, although yielding insights, considerably decreases sample size. Our method may be particularly applicable for pathogens with a relatively high seroprevalence, and for species in which it is relatively easy to collect biological samples, such as species of conservation concern where there is routine monitoring, or species which are hunted for sport.

Disease‐induced fitness consequences in the wild can be difficult to establish as appropriate data is often limited. Also, the frequency and nature of different transmission routes for wild animals, such as pumas, can be unclear, due to the difficulty in observing or simulating transmission. This study presents a method of assessing vital parameters for pathogen transmission and the impact of a disease on host mortality, using age‐prevalence data. Our approach to estimate the age‐specific FOI is a way of revealing the effects of age on infection risk, which can identify those subpopulations most at risk of infection, highly useful for disease management. It also offers insights into the plausibility of different transmission routes. In our study system, aggressive encounters between pumas seem to be one important mode of FIV transmission, supported by the result that males generally have a higher FOI than females. Our findings also suggest that transmission from mothers to cubs is a more common means of puma FIV transmission than previously thought.

## CONFLICT OF INTEREST

None declared.

## Data Availability

Data associated with this manuscript are archived in the Dryad Digital Repository: https://doi.org/10.5061/dryad.59dh3cm

## APPENDIX A

Here, we consider the case when *λ* is constant and *μ* ≠ 0. The following equations describe disease acquisition:

(A1) $$\frac{dI}{da}=\lambda S-\mu I$$

(A2) $$\frac{dS}{da}=-\lambda S$$

with *S* the proportion of susceptible animals, *I* the proportion of infected animals, and *a* the age. Equation (A2) gives $S=e^{-\lambda a}$. So we get

$$\begin{array}{c} \frac{dI}{da}=\lambda e^{-\lambda a}-\mu I\\ \Rightarrow e^{\mu a}I=\lambda \int e^{\mu a}e^{-\lambda a}da\\ =\lambda \int e^{(\mu -\lambda )a}da\\ =\frac{\lambda e^{(\mu -\lambda )a}}{\mu -\lambda }+C \end{array}$$

where *C* is a constant of integration. Given that *I* = 0 when *a* = 0, C=-(λ/μ-λ). Thus

$$\begin{array}{c} I=\frac{\lambda e^{(\mu -\lambda )a}}{\left(\mu -\lambda \right)e^{\mu a}}-\frac{\lambda }{\left(\mu -\lambda \right)e^{\mu a}}\\ =\frac{\lambda [e^{(\mu -\lambda )a}-1]}{\left(\mu -\lambda \right)e^{\mu a}}. \end{array}$$

Now, the ratio

$$\frac{I}{I+S}$$

represents the prevalence of infection in the population. Using the above expressions for *S* and *I*, we get

(A3) $$\begin{array}{c} \frac{I}{I+S}=\frac{\lambda [e^{(\mu -\lambda )a}-1]}{(\mu -\lambda )e^{\mu a}}\times \frac{\left(\mu -\lambda \right)e^{\mu a}}{\lambda e^{(\mu -\lambda )a}-\lambda +e^{(\mu -\lambda )a}\left(\mu -\lambda \right)}\\ =\frac{\lambda [e^{(\mu -\lambda )a}-1]}{-\lambda +\mu e^{(\mu -\lambda )a}}\\ =\frac{\lambda [1-e^{(\mu -\lambda )a}]}{\lambda -\mu e^{(\mu -\lambda )a}}. \end{array}$$

The next step is to fit Equation (A3) to the data on prevalence of FIV in the puma population, to yield estimates of parameters *λ* and *μ*.

**Table A1:** AICc values for each model for data from all three sites and both sexes used together

Force of infection *λ*(*a*)	AICc	
	*μ* = 0	*μ* ≠ 0
Constant	*270.68*	*269.42*
Weibull	272.72	271.48
Log‐logistic	**269.31**	271.53
Polynomial, *k* = 1	272.72	*271.02*
Polynomial, *k* = 2	274.77	272.58

Note

The lowest AICc value is shown in bold, and any others that are <2 from the lowest are italicized.

**Table A2:** AICc values for each model for data separated according to sex

Force of infection *λ*(*a*)	Females		Males	
	AICc		AICc	
	*μ* = 0	*μ* ≠ 0	*μ* = 0	*μ* ≠ 0
Constant	**131.95**	134.02	136.53	*134.06*
Weibull	134.02	136.12	138.61	136.15
Log‐logistic	134.31	136.42	**134.04**	136.18
Polynomial, *k* = 1	134.02	135.81	138.61	136.14
Polynomial, *k* = 2	136.07	137.80	140.74	138.29

Note

The lowest AICc value for females and for males is shown in bold, and any others that are <2 from the lowest are italicized.

## APPENDIX B

Octave code for optimization routine:

[para,lik,info,iter]=sqp(init,@h,[ ],[ ],low,up,maxiter,tol)

### OUTPUT

para = maximum likelihood parameter estimates

lik = best negative log‐likelihood value

info = information about the optimization

iter = number of iterations

### INPUT

init = initial guess for parameter values

@h = function handle pointing to what is being minimized (negative log‐likelihood)

low = lower bounds for the parameter values

up = upper bounds for the parameter values

maxiter = maximum number of iterations (set to 1000; default 100)

tol = tolerance for stopping criteria (set to 0.0000000001)
